# Immunogenicity of a secreted, C-terminally truncated, form of bovine viral diarrhea virus E2 glycoprotein as a potential candidate in subunit vaccine development

**DOI:** 10.1038/s41598-022-26766-y

**Published:** 2023-01-06

**Authors:** Yi Ting Lo, Martin D. Ryan, Garry A. Luke, Wan Chen Chang, Hsing Chieh Wu

**Affiliations:** 1 International Degree Program in Animal Vaccine Technology, International College, National Pingtung University of Science and Technology, No. 1, Shuefu Rd., Neipu, Pingtung 91201 Taiwan; 2 Biomedical Sciences Research Complex, School of Biology, University of St Andrews, St Andrews, Scotland, UK; 3 Graduate Institute of Animal Vaccine Technology, College of Veterinary Medicine, National Pingtung University of Science and Technology, Neipu, Pingtung Taiwan

**Keywords:** Protein vaccines, Applied microbiology

## Abstract

Both current live, attenuated, and killed virus vaccines for bovine viral diarrhea virus (BVDV) have their limitations. Here, we report the development of a BVDV subunit vaccine by (i) the expression of a secreted form of a recombinant E2 glycoprotein using BHK21 cells and (ii) determination of the immune responses in mice. The E2 glycoprotein was modified by deletion of the C-terminal transmembrane anchor domain and fusion to a V5 epitope tag. This allowed detection using anti-V5 monoclonal antibodies together with simple purification of the expressed, secreted, form of E2 from the cell media. Furthermore, we genetically fused green fluorescent protein (GFP) linked to E2 via a *Thosea asigna* virus 2A (T2A) ribosome skipping sequence thereby creating a self-processing polyprotein [GFP-T2A-BVDV-E2^trunk^-V5], producing discrete [GFP-T2A] and [E2^trunk^-V5] translation products: GFP fluorescence acts, therefore, as a surrogate marker of E2 expression, BALB/c mice were inoculated with [E2^trunk^-V5] purified from cell media and both humoral and cellular immune responses were observed. Our antigen expression system provides, therefore, both (i) a simple antigen purification protocol together with (ii) a feasible strategy for further, large-scale, production of vaccines.

## Introduction

Bovine viral diarrhea virus (BVDV) is a major infectious agent in cattle, pigs, and other ruminants; it causes bovine viral diarrhea and mucosal disease, leading to various clinical outcomes ranging from mild illness to acute and severe life-threatening disease^1^. Clinical signs of BVDV infection include fever, depression, anorexia, mucosal erosions, purulent ocular and nasal discharge, coughing, rumen atony, diarrhea, abortion, and decreased milk yield^2^. BVDV infection also causes immunosuppression, thereby increasing susceptibility to bacterial or viral pneumonia^3^. In animals with BVDV-induced immunosuppression, exposure to other pathogens can greatly increase their morbidity and mortality, with a corresponding detrimental economic impact on the beef and dairy industries^4,5^.

The BVDV genome is a single-stranded positive-sensed RNA of approximately 12.3 kb. BVDV belongs to the genus *Pestivirus* in the family *Flaviviridae*, along with border disease virus (affecting sheep) and classical swine fever virus^6^. BVDV comprises two main genotypes: BVDV-1 (*Pestivirus* A, divided into 22 subtypes from BVDV-1a to BVDV-1v) and BVDV-2 (*Pestivirus* B, divided into four subtypes from BVDV-2a to BVDV-2d). Another atypical pestivirus known as HoBi-like virus was tentatively identified as BVDV-3, which has four subtypes^7^. Each BVDV has two biotypes, cytopathic and noncytopathic, according to the effects of the virus grown on cultured cells. The noncytopathic strain does not damage infected cells^8^. It can infect the developing fetus if cows are exposed to the virus during early gestation. Moreover, BVDV is perpetuated in the herd through the production of persistently infected (PI) calves^9^, since they become immunotolerant to the virus and shed large amounts of virus throughout their lives. Therefore, PI animals are regarded as a major source for spreading BVDV^10^. The most effective measures in BVDV prevention programmes include biosecurity measures, eradication of PI cattle, and vaccination^5,11^.

Current vaccines against BVDV are multivalent modified-live virus vaccines and inactivated virus vaccines^11–14^. Commercially available live, attenuated, and inactivated vaccines have limited safety and efficacy: the modified-live virus vaccine can revert to virulence and lead to PI calves, whereas the inactivated virus vaccine has a short duration of protection and cannot stimulate a cellular immune response^15^. Therefore, a safe and efficacious subunit BVDV vaccine is required for long-term protection of cattle against BVDV.

The transmembrane protein E2 is one of the BVDV structural proteins, anchored in the viral membrane via the C-terminal domain. As a major immunogenic glycoprotein, E2 is involved in virus fusion with host cells and contains major antigenic sites that can elicit neutralizing antibodies during infection with BVDV^16,17^. Therefore, E2 is a prime candidate for subunit vaccine design. In this study, we developed a eukaryotic expression system to express recombinant E2 glycoprotein that was cloned with the transmembrane anchor domain deleted, enabling this E2 truncated glycoprotein to be secreted into the tissue culture media after mammalian cell transfection. Recombinant E2 glycoprotein was produced in mammalian cells to ensure the correct post-translational modifications, and the correct glycosylation patterns were observed to be immunogenic in mice. The sequences encoding the transmembrane domain–deleted form of E2 were genetically fused to the V5 epitope tag for (i) protein detection by Western blotting and (ii) purification of the expressed truncated E2 from the cell media. Furthermore, we used the *Thosea asigna* virus 2A (T2A) self-cleaving sequence to link the truncated E2 gene and green fluorescent protein (GFP). The 2A peptides are small and can mediate cleavage efficiently for co-expression of multiple genes^18,19^. The GFP was used as a surrogate marker for E2 protein expression, this marker being easily visualized by non-invasive, live-cell, fluorescence microscopy following transfection of Baby hamster kidney (BHK)-21 cells. We also evaluated the immunogenicity of secreted C-terminal truncated E2 glycoproteins in mice through a range of immune responses, namely the titre of antigen-specific antibodies in serum, lymphocyte proliferation, subpopulation in splenocytes, and concentration of various cytokines.

## Results

### Molecular cloning of the truncated form of BVDV E2 glycoprotein

**Figure 1:**
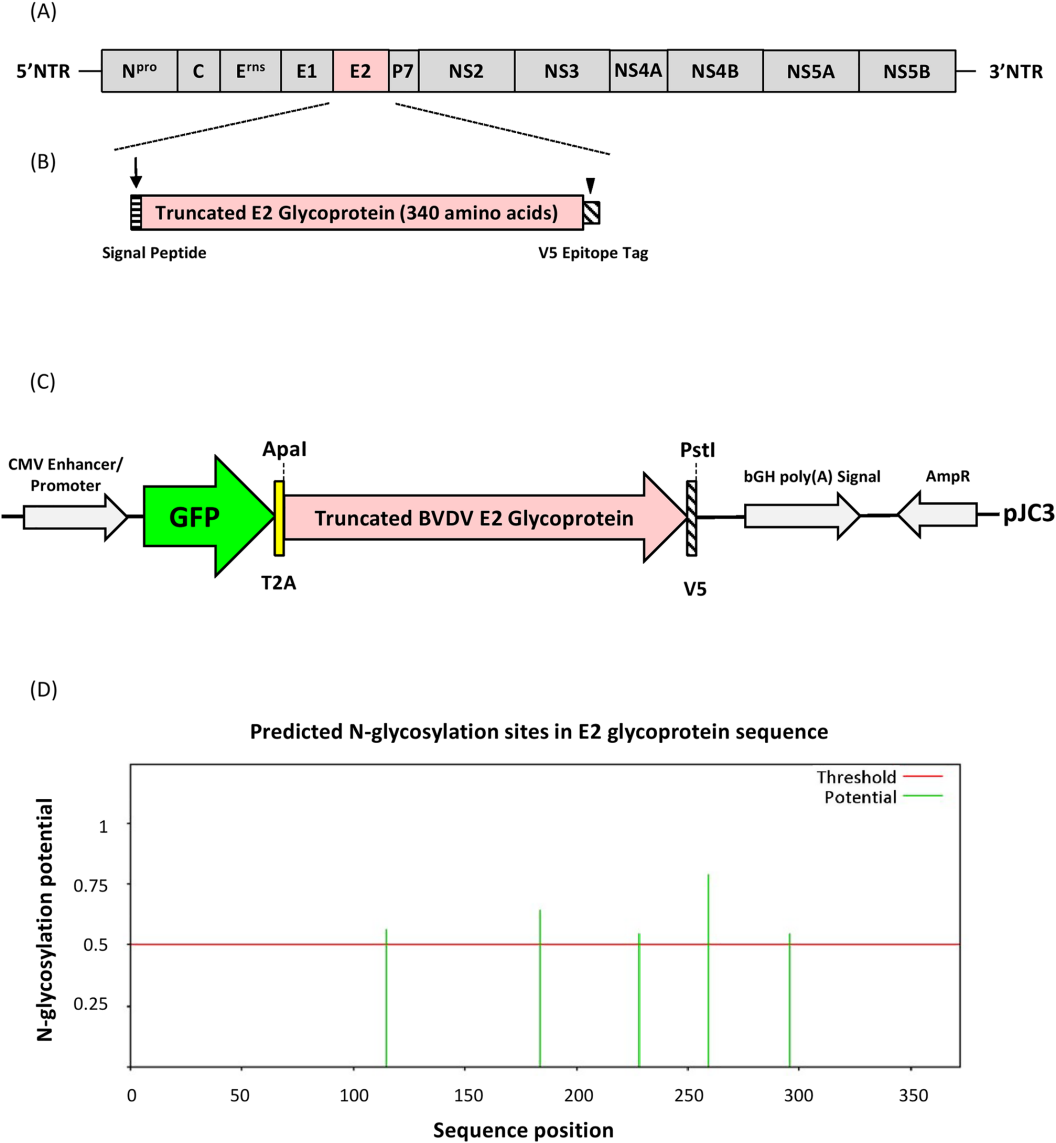
Genome structure of BVDV and synthesis of the region encoding the E2 glycoprotein. BVDV genome organization (not to scale) and polyprotein processing (**A**). Cloning strategy and synthesis of genes encoding the C-terminally truncated (transmembrane anchoring domains deleted) forms of the E2 glycoprotein (boxed areas) are shown, together with the signal peptide (horizontal hatching area) and the C-terminal V5 epitope tag (slash area) (**B**). Cloning of truncated form of E2 glycoprotein into pJC3 expression vector. Plasmid pJC3 encodes a [GFP-T2A-mCherry] polyprotein encoded by a single open reading frame. Transcription is driven by the human cytomegalovirus enhancer/promoter and the mRNA polyadenylated by the bovine growth hormone polyadenylation signal. Sequences encoding the truncated form of E2 glycoprotein were excised by restriction with ApaI and PstI from the pGEM-T Easy Vector, then inserted to pJC3, similarly restricted, to remove mCherry FP and create plasmid [GFP-T2A-BVDV-E2 trunk] (**C**). The NetNGlyc-1.0 server (https://services.healthtech.dtu.dk/service.php?NetNGlyc-1.0) was used to predict N-glycosylated sites from full-length E2 glycoproteins. An N-glycosylation potential of > 0.5 was the cut-off value for sequences (**D**).

The synthesized C-terminally truncated form of the BVDV E2 gene (Fig. 1A and B) was initially cloned into the pGEM-T Easy Vector system and verified by automated Sanger sequencing before being excised (using ApaI and PstI) from this vector and being transferred to the pJC3 expression vector (Fig. 1C). The NetNGlyc-1.0 program (https://services.healthtech.dtu.dk/service.php?NetNGlyc-1.0)^20^ was used to predict N-glycosylation sites from the full-length BVDV E2 glycoprotein. This program predicts that the full-length BVDV E2 glycoprotein has 5 potential N-glycosylation sites (N115, N184, N228, N259, 296) (Fig. 1D).

### Detection of secreted and cell-associated protein

**Figure 2:**
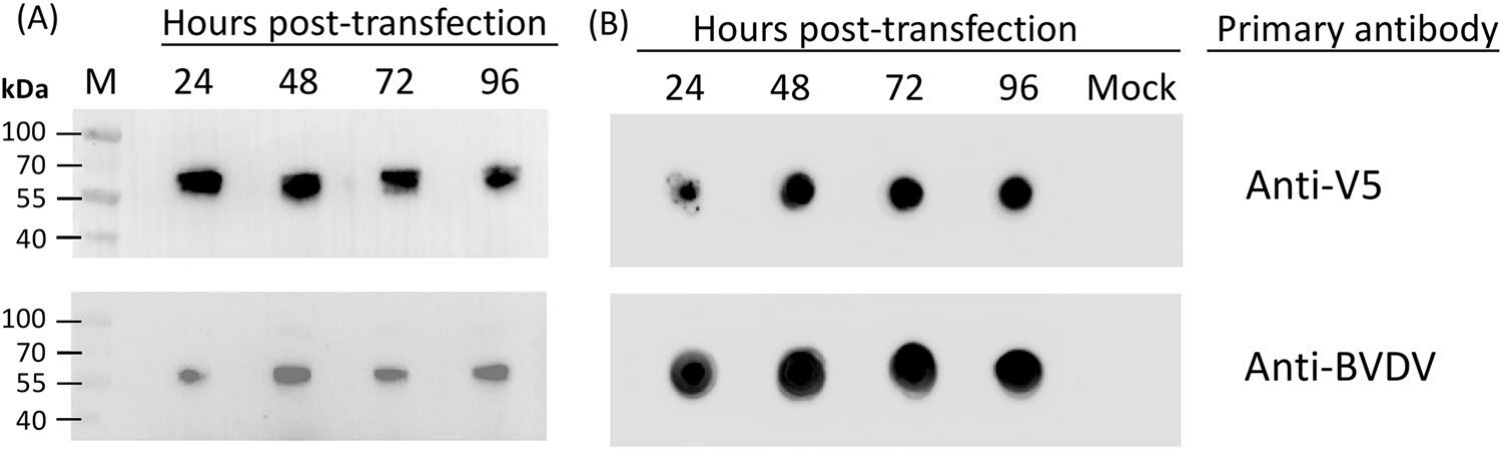
Evaluation of truncated E2 glycoprotein expression. (**A**) Western blot analyses of transfected cell extracts. Samples of cellular extracts were prepared and analyzed (12% SDS–PAGE) prior to the transfer, as described in methods. (**B**) Immuno-dot blot analyses of the cell media. Tissue culture media from BHK-21 cells transfected with truncated E2 glycoprotein at the indicated time points. Membranes were probed with the mouse anti-V5 monoclonal antibody (1:8000) or mouse anti-BVDV antiserum (1:4000) as primary antibody. Lane M: prestained protein marker, lane mock: untransfected BHK-21 cells.

To determine if the C-terminally truncated form of the BVDV E2 glycoprotein was secreted into the cell culture media, immuno-dot blot assays were employed on the media using a monoclonal antibody against the V5 epitope tag or mouse anti-BVDV antiserum present at different times post-transfection. Western blot analysis of transfected BHK-21 cell extracts indicated maximal expression of the truncated E2 glycoprotein at 48 h. The protein band observed at ~ 55 kDa is somewhat diffuse, consistent with the degree of heterogeneity in molecular mass which arises from protein glycosylation (Fig. 2A). The immune dot blots indicated that the truncated E2 glycoprotein was secreted into the media and recognized by the V5 epitope tag and BVDV antiserum (Fig. 2B); this secreted form was stable in the cell media for the duration of the experiment (96 h), consistent with the Western blot data.

### Humoral immune response

**Figure 3:**
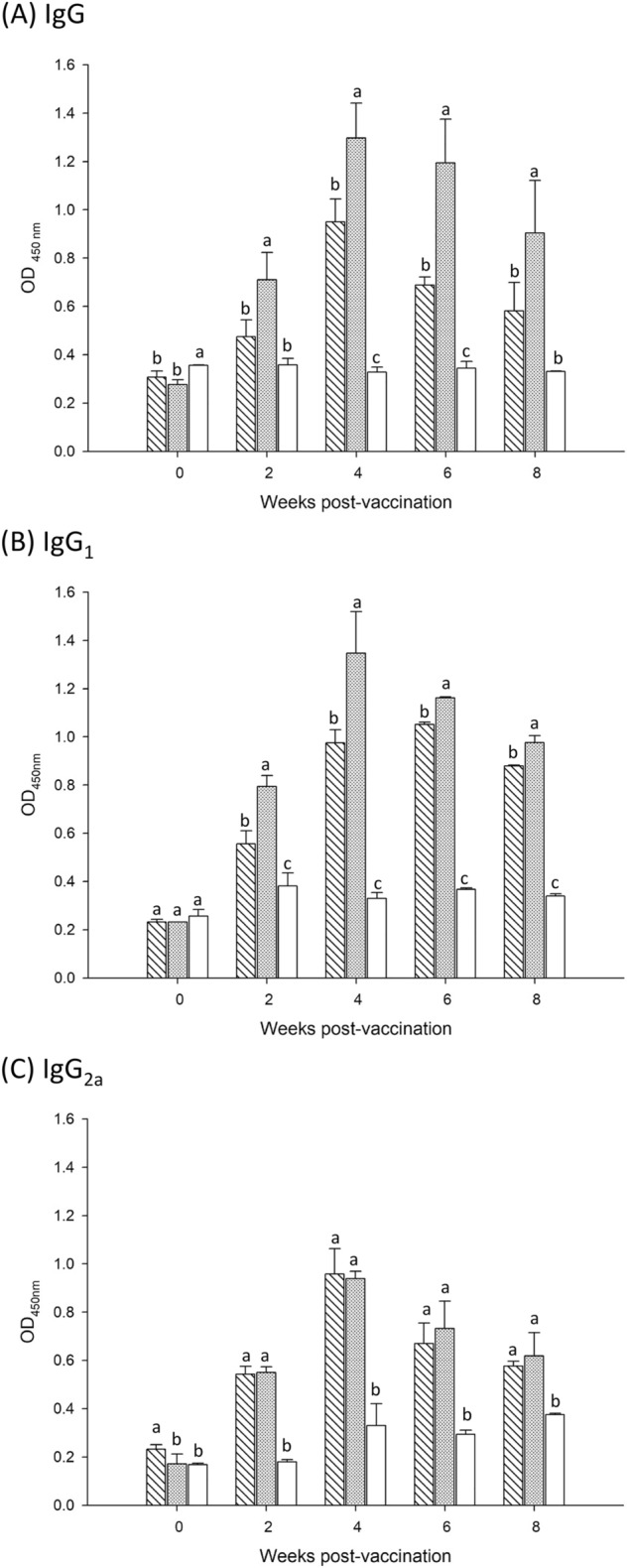
ELISA analysis of BVDV-specific antibody responses induced by truncated E2 glycoprotein: (**A**) total IgG, (**B**) IgG_1_, and (**C**) IgG_2a_. Antibody responses in mice were assessed at 0, 2, 4, 6, and 8 weeks after intraperitoneally immunization with 5 or 35 μg of truncated E2 glycoprotein or PBS. Mice were injected twice at weeks 0 and 2. Data are presented as mean absorbance values ± standard deviations. Different letters (a, b, c) on the bar indicate significant (*P* < 0.05) between-group differences at the same time point using Duncan’s test. () Group I: 5 μg of truncated E2 glycoprotein; () Group II: 35 μg of truncated E2 glycoprotein; () Group III: PBS.

The serum concentrations of BVDV-specific antibodies of different subtypes (IgG, IgG_1_, and IgG_2a_) were determined using ELISA. After the first vaccination, the total IgG antibody titre in the high-dose group was significantly (*P* < 0.05) higher than that in the low-dose group until week 8 (Fig. 3A). Additionally, truncated E2 glycoprotein stimulated the simultaneous production of IgG_1_ (Fig. 3B) and IgG_2a_ (Fig. 3C) antibodies. Compared with the control group, IgG1 level was increased by approximately 3.5-fold and IgG_2a_ level by approximately 2.5-fold, indicating upregulation of the IgG subtypes associated with T cell-dependent immunity.

### Evaluation of lymphocyte proliferation in response to truncated E2 glycoprotein stimulation

**Figure 4:**
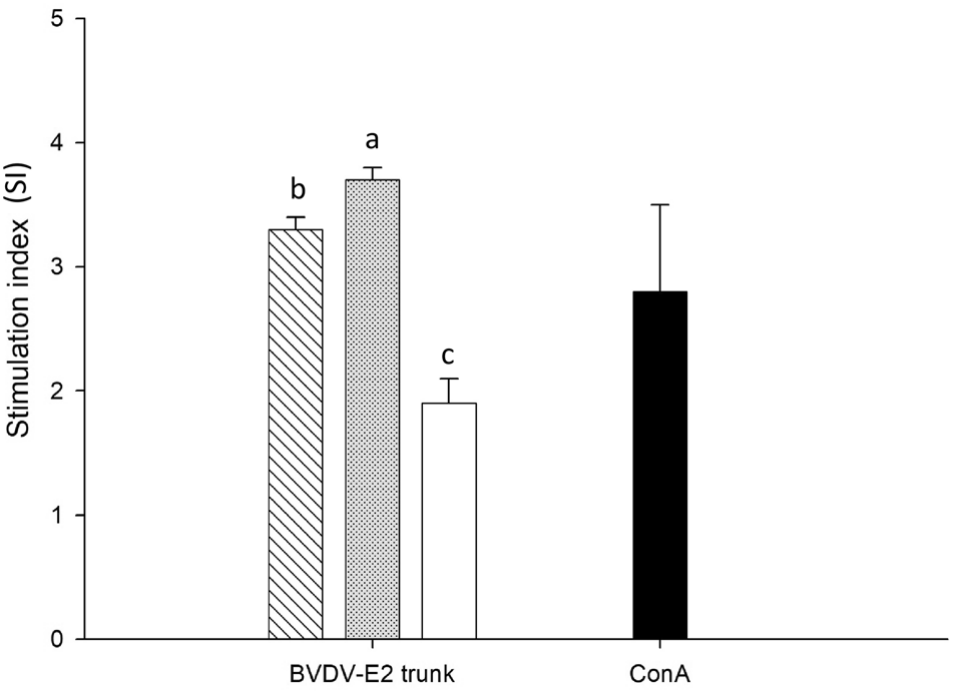
Stimulation of mice lymphocyte proliferation by truncated E2 glycoprotein. Stimulation index (SI) was calculated by dividing the mean OD value of stimulated wells by that of the control wells (media only). The data are presented as the mean ± standard deviations of three individual experiments. Treatments with different letters (a, b, c) on the bar indicate significant (*P* < 0.05) differences. () Group I: 5 μg of truncated E2 glycoprotein;() Group II: 35 μg of truncated E2 glycoprotein;() Group III: PBS;() Positive control: ConA.

To evaluate the cell-mediated immune response, mice splenocytes were isolated and restimulated in vitro with 5 μg of truncated E2 glycoprotein. Lymphocyte proliferation was evaluated to represent the stimulation index (SI). As Fig. 4 illustrates, the T lymphocyte proliferative activity in the high-dose group was significantly (*P* < 0.05) higher than those in low-dose and control groups. No effect on lymphocyte proliferation was noted in the control group, which showed that the truncated E2 glycoprotein can induce a cell-mediated immune response in vivo.

### Evaluation of CD4^+^ and CD8^+^ T cell responses

**Figure 5:**
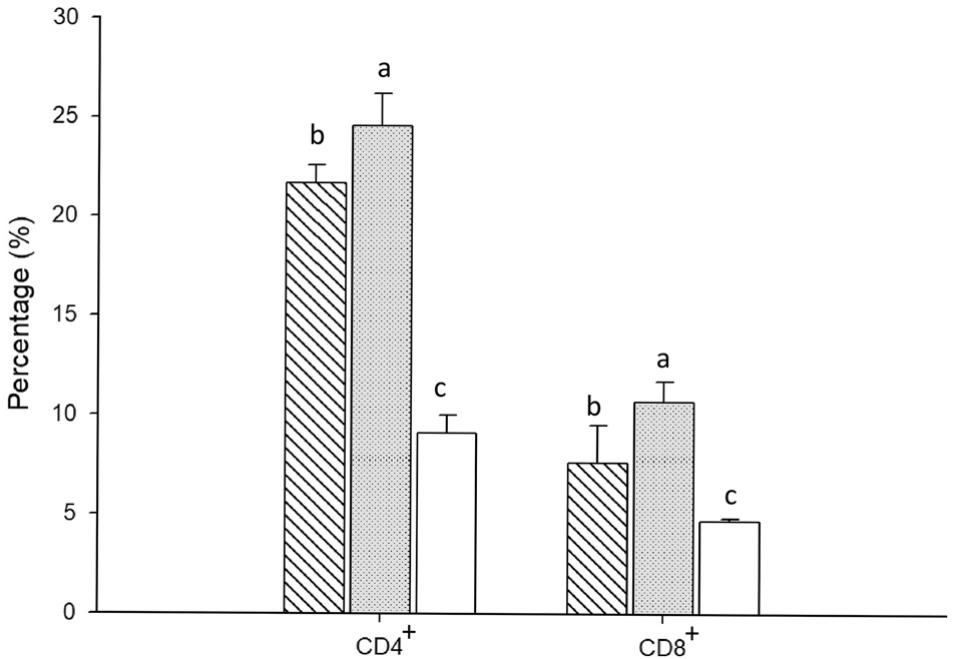
Flow cytometric analysis of the percentage of CD4^+^ and CD8^+^ T lymphocytes in spleen. Mice were immunized intraperitoneally twice at weeks 0 and 2 with truncated E2 glycoprotein, and mice in the control group were immunized with PBS. Mice splenocytes were isolated at 4 weeks post-immunized and stained with anti-mouse CD4^+^ and CD8^+^ monoclonal antibody. Data are presented as the mean ± standard deviations of the mice in the same treatment (n = 4). Different letters (a, b, c) on the bar indicate significant (*P* < 0.05) between-group differences at the same time point.() Group I: 5 μg of truncated E2 glycoprotein;() Group II: 35 μg of truncated E2 glycoprotein;() Group III: PBS.

To determine the phenotype of the T cell subpopulation in spleen lymphocytes, flow cytometry was conducted, with single labelling for defining different subpopulations. At 4 weeks post-immunization, the percentage of increase in CD4^+^ T lymphocytes in the spleen was significantly higher in the high-dose group (24.6% ± 1.6%, *P* < 0.05) than in the other groups, and the percentage of increase in CD8^+^ T lymphocytes in the spleen was significantly higher in the high-dose group than in the low-dose group (5 μg) (10.7% ± 1.0% vs. 7.7% ± 1.9%, *P* < 0.05; Fig. 5).

### Serum IL-12, IFN-γ, and IL-4 levels

**Figure 6:**
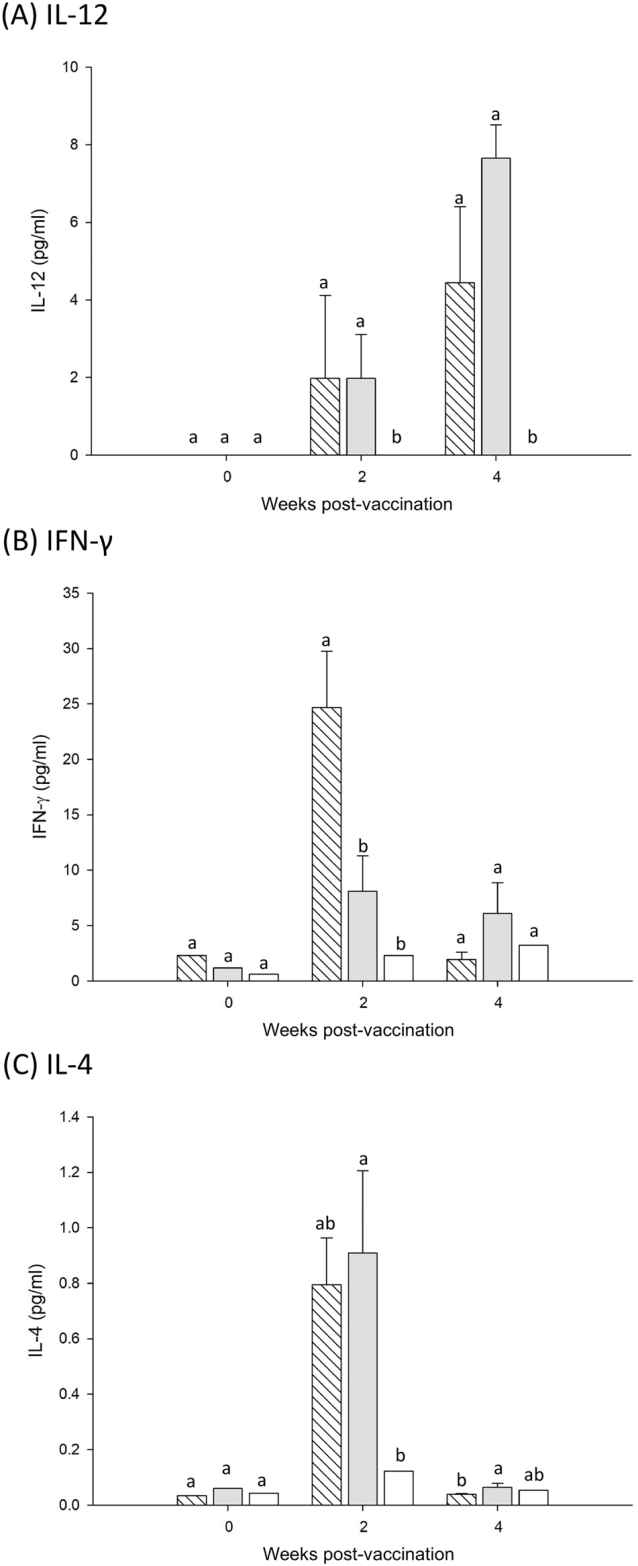
Concentration of cytokines (**A**) IL-12, (**B**) IFN-γ, and (**C**) IL-4 in mice immunized with 5 or 35 μg of truncated E2 glycoprotein or PBS. Data are presented as mean ± standard deviations of the sera concentration of each cytokine determined using commercial ELISA kits. Different letters (a, b, c) on the bar indicate significant (*P* < 0.05) between-group differences at the same time point.() Group I: 5 μg of truncated E2 glycoprotein;() Group II: 35 μg of truncated E2 glycoprotein;() Group III: PBS.

To further characterize the immune response polarization, the concentrations of Th1 cytokines, IL-12 (Fig. 6A) and IFN-γ (Fig. 6B), or a Th2 cytokine, IL-4 (Fig. 6C), in the serum from immunized mice were measured on weeks 0, 2, and 4. Unlike the sera of the control group, the sera of mice in both study groups contained IL-12, IFN-γ, and IL-4. These results demonstrate that truncated E2 glycoproteins can effectively elicit both specific Th1 and Th2 responses.

## Discussion

The control of BVDV infection in cattle relies on biosecurity measures, identification and removal of PI cattle, and vaccination. PI calves are the principal transmitters of BVDV since they become immunotolerant to the virus and shed large amounts of virus throughout their lives. Therefore, vaccinations now help provide immune populations; immunity is particularly vital for the breeding females in the herd^1^. In recent years, many studies have attempted to develop more efficacious BVDV vaccines. Live, attenuated, vaccines have been widely used against BVDV, inducing strong humoral and cell-mediated immune responses with strong fetus protection^21,22^. Live, attenuated, vaccines based on the noncytopathic strain are generally not recommended for the vaccination of pregnant animals since this strain can cross the placenta and infect the fetus. Studies have attempted to overcome the safety problem of developing a mutant virus by deleting the N^pro^ gene and inactivating the endoribonuclease activity of the structural glycoprotein E^rns^^22,23^, but the vaccine virus could still cross the placenta^24^. Inactivated vaccines are safe during pregnancy^25–27^, but their efficacy is limited because they do not induce a cellular immune response^28,29^. No significant cross-protection against different virus strains have been reported^30,31^. Therefore, the development of subunit vaccines is an effective approach. Additionally, antigens expressed in different virus strains are potential vaccine candidates; these vaccines might trigger cross-protection against different BVDV strains. In the present study, we cloned and expressed a truncated E2 glycoprotein. The E2 glycoprotein is the most divergent antigen among BVDV types 1 and 2 and HoBi-like pestivirus, and it can elicit neutralizing antibodies^16,17^.

The main objectives of the present study were (i) to clone a secreted truncated form of BVDV E2 glycoprotein, (ii) to use mammalian cell culture as our antigen expression platform, and (iii) to evaluate the immune effects of the vaccine in mice. The construction strategy we applied in this study was to generate a genetic fusion of C-terminal truncated (deleted transmembrane anchor) E2 glycoprotein with GFP linked via a T2A ribosome skipping sequence to create a self-processing polyprotein (GFP-T2A-BVDV-E2 trunk). This strategy does not affect the function of each protein downstream of T2A^18,19,32^. Moreover, the advantages of this strategy are (i) the ease of identifying and quantifying GFP fluorescence live-cell signal intensities by using assays and (ii) subsequent fluorescence activated cell sorting of stably transfected cells. Subunit vaccines often depend on a single protein to generate an effective immune response within the vaccinated host; as such, both the quantity and antigenic authenticity of the recombinant protein are crucial^33^. Therefore, our truncated E2 glycoprotein was expressed in mammalian cell lines to produce more viral-type authentic protein folding and complex glycosylation that affects various protein functions, including folding, stability, activity, authentic antigenicity, transport, and immune response. Our data indicated that the truncated E2 glycoprotein was successfully secreted into the tissue culture media (Fig. 2B) and on-going expression detect within the cell (Fig. 2A), consistent with our previously reported findings^32^. The Western blot analyses of cell extracts after transfection of truncated E2 glycoprotein revealed a diffuse, rather than a discrete band, of the predicted molecular weight (55 kDa), characteristic of glycosylation (Fig. 2A). In addition, the truncated E2 glycoprotein exhibited antigenicity, as demonstrated in immune blotting by using mouse anti-BVDV antiserum (Fig. 2).

Because the quality of the immune response is crucial for vaccine efficacy, we investigated the immune response following immunization with the truncated E2 glycoprotein vaccine. When whole-virus BVDV was used as an ELISA coating antigen, the titre of total IgG was significantly higher in the vaccination groups (truncated E2 glycoprotein) compared with the control group after immunization (Fig. 3A). In addition, the truncated E2 glycoprotein mediated both BVDV-specific IgG1 (Th2) (Fig. 3B) and IgG2a (Th1) (Fig. 3C) antibody responses. Moreover, the vaccine-immunized mice exhibited IgG1 predominance, which is related to Th2 shift response. In parallel, the IL-4 levels in the vaccination groups were significantly higher than those in the control group (Fig. 6C). IL-4 is a Th2 type representative cytokine that is associated with B cell proliferation, differentiation, and maturity, and regulates antibody production^34^. Taken together, these results reveal that the truncated E2 glycoprotein is effective for inducing a specific humoral immune response.

Th1 cells are involved in cellular immunity and secrete the effector Th1-type cytokine. Thus, the Th1 cytokine level and T lymphocyte proliferative activity can reflect the basic status of cellular immunity^35^. Our data indicated that the Th1 type cytokine levels (IL-12 and IFN-γ) in the vaccination groups after immunization were significantly higher than in the control group (Fig. 6A and B). However, no significant difference was observed between the low-dose and high-dose groups. Notably, we observed a markedly enhanced SI in the restimulated spleen T lymphocytes of mice immunized with truncated E2 glycoprotein (Fig. 4). Furthermore, the percentages of both CD4^+^ and CD8^+^ T cells in the spleen of immunized mice were significantly increased, although CD4^+^ was slightly higher than CD8^+^ (Fig. 5), indicating strong activation of T cells. CD4^+^ T cells secrete a spectrum of cytokines and are critical to the activation of both humoral and cellular immunity, whereas CD8^+^ T cells are associated with the cytokine activity of T lymphocytes for eliminating intracellular pathogens ^36^. These findings suggest that the truncated E2 glycoprotein can induce a cellular immune response in mice.

## Conclusions

In this study, our antigen expression strategy yielded a secretory viral-type authentic recombinant protein and provides a feasible strategy for large-scale production of vaccines; further simple purification is possible with a V5 epitope tag. Moreover, truncated E2 glycoproteins were found to induce both humoral and cellular immune responses. Notably, we did not apply an additional adjuvant in the vaccine formulation, highlighting the favourable immunogenicity of the truncated E2 glycoprotein. Our construction strategy enabled us to obtain high-quality antigen expression, and our evaluation confirmed the efficacy of the vaccine. These promising results can serve as a basis for further research. In the future, we will carry out field trials in cattle to have a more detailed investigation of the effectiveness and comprehensively evaluate the potential of this subunit vaccine against BVDV.

## Methods

### Plasmid DNA constructions

The transmembrane domain of the BVDV E2 glycoprotein was predicted using the TMHMM-2.0 program (https://services.healthtech.dtu.dk/service.php?DeepTMHMM). To our knowledge, only BVDV type 2 strain has been reported in cattle herds in Taiwan. Therefore, the C-terminally truncated form of the BVDV E2 gene (position 2462–3481 nucleic acid) was synthesized using a sequence isolated from cattle in China (strain XJ-04, GenBank nucleotide sequence accession no. FJ527854) that also encoded (i) a Tobacco Etch Virus protease cleavage site (-ENLYFQG-) that can be used to remove both (ii) a short linker sequence (-GSDQTENSG-) and (iii) the V5 epitope tag (-GKPIPNPLLGLDST-_COOH_) (Fig. 1A and B). The synthesized BVDV E2 gene (Eurofins Genomics, Ebersberg, Germany) was cloned into the pGEM-T Easy Vector (Promega, Madison, WI, USA), as per the manufacturer’s instructions. All clones were verified through automated Sanger sequencing (GATC Biotech, Cambridge, UK).

The encoded sequence of BVDV *E2* gene was digested with ApaI and PstI (New England Biolabs, Hitchin, UK) and cloned into the pJC3 expression vector, similarly restricted (Fig. 1C). The pJC3 expression vector was pcDNA3.1-based, with T2A linking GFP and monomeric cherry fluorescent protein (mCherry FP) genes^37,38^. This yielded a clone encoding the truncated form of the E2 glycoprotein (GFP-T2A-BVDV-E2 trunk).

### Cells and virus

BHK-21 cells (BCRC no. 60041) and Madin–Darby bovine kidney (MDBK) cells (BCRC no. 60126) were obtained from the Bioresource Collection and Research Center, Taiwan, and propagated in Eagle’s minimum essential medium (Invitrogen, NY, USA) supplemented with heat-inactivated 10% fetal bovine serum (FBS; Invitrogen, Carlsbad, CA, USA) in 5% CO_2_ at 37 °C.

In this study, we used the BVDV type 2 strain BVDV/TW 2014 obtained from the Large Animal Hospital, National Pingtung University of Science and Technology (NPUST), Taiwan^39^. The virus was cultured in MDBK cells. BVDV titres were determined using the Reed and Muench^40^ method and expressed as the fluorescent antibody infectious dose FAID_50_/mL^39,41^. In brief, a virus sample (100 μL) and MDBK cells (100 μL of 1 × 10^5^ cells/mL) were co-cultured in 96-well plates for 6 days at 37 °C in 5% CO_2_ atmosphere. Buffered Formalde-Fresh solution (Thermo Fisher Scientific, Waltham, MA, USA) was used to fix the cells, and a 1:5 dilution of anti-BVDV fluorescence-conjugated polyclonal antiserum (VMRD, Pullman, WA, USA) was used for virus detection. Fluorescent wells were observed using a fluorescence microscope (Olympus CKX41, Olympus Co. Ltd., Tokyo, Japan).

### Detection of secreted protein using immuno-dot blot assay

To detect the secreted protein, plasmid DNA was transfected into BHK-21 cell monolayers (1 × 10^7^ cells per T-175 flask, 70%–80% confluent) by using PolyJet In Vitro DNA Transfection Reagent (SignaGen Laboratories, Ijamsville, MD, USA) as per the manufacturer’s instructions. The media and cell extract were collected at 24, 48, 72, and 96 h post-transfection into the BHK-21 cells. Cell culture media were collected and clarified through centrifugation (2000×*g* at 4 °C for 15 min), and the supernatant was decanted to a new tube before being stored at − 20 °C. Supernatants were thawed on ice before being spotted onto a nitrocellulose membrane (0.2 µm; Bio-Rad, Hercules, CA, USA), where they were analyzed using dot blot assay with a monoclonal antibody against the V5 epitope tag (1:8000; MBL International, Nagoya, Japan) or mouse anti-BVDV antiserum (1:4000)^39^ as the primary antibody and horseradish peroxidase (HRP)-labelled goat anti-mouse IgG as the secondary antibody (1:8000; KPL, Gaithersburg, MD, USA). The signal was developed using the Clarity Western ECL Substrate (Bio-Rad).

### Western blot analysis of cell-associated protein

At 24, 48, 72, and 96 h post-transfection, the BHK-21 cells were washed, and the cells lysed using RIPA lysis and extraction buffer (Thermo Fisher Scientific, Rockford, IL, USA) containing EDTA-free protease inhibitor cocktail (cOmplete; Roche Diagnostics, Burgess Hill, UK), as per the manufacturer’s instructions. The cell lysate was then analyzed using SDS–PAGE and electroblotted onto polyvinylidene difluoride transfer membranes (Immobilon-P; Merck Millipore, Tullagreen, Ireland). After blocking with 5% Difco Skim Milk (w/v, BD, Le Pont de Claix, France), the membranes were probed overnight at 4 °C with a primary antibody that recognised the V5 epitope tag (1:8000; MBL International) or mouse anti-BVDV antiserum (1:4000)^39^. After washing three times in Tris-buffered saline containing Tween 20, membranes were incubated for 1 h at room temperature with goat anti-mouse IgG-HRP as the secondary antibody (1:8000; KPL) and imaged using the G:BOX imaging system and analysis software (Syngene, Frederick, MD, USA).

### Quantification of secreted form BVDV E2 glycoprotein and vaccine preparation

To quantify the truncated E2 glycoprotein, the media were collected 48 h after transfection of the BHK-21 cells. The clarified supernatant was applied to the V5-tagged Protein Purification Kit Version 2 (MBL International) in accordance with the manufacturer’s protocol. The eluted protein fractions containing the truncated E2 glycoprotein were pooled and concentrated using an Amicon ultracentrifugal filter with a pore size of 30 kDa (Merck Millipore, Burlington, MA, USA). Protein estimation was performed using the Pierce BCA Protein Assay Kit (Thermo Fisher Scientific, Rockford, IL, USA).

The vaccine used in this study was a non-adjuvant vaccine, containing a purified truncated form of E2 glycoprotein divided into low (5 µg) and high (35 µg) concentrations per dose. The vaccine antigen concentration was based on previous dose-dependent experiment^32^. The vaccines contained no viable microorganisms, according to the results of sterility test.

### Vaccination and serum sample collection

The mouse experiments were approved by the Animal Care and Use Committee of the NPUST and conducted following the ethical rules and laws of the university (IACUC Approval No. 110-046). Mice used in this study were bred and housed in an aseptic environment to protect them from opportunistic infections. The mice were maintained under a light–dark cycle of 12:12 h at a constant temperature (22 ± 1 °C) and a relative humidity of 50% ± 10%; all animals had ad libitum access to food and water. All efforts were made to minimize animal suffering, and the animals were humanely euthanized at the end of the study period. The study was carried out in compliance with the ARRIVE guidelines.

Twenty-seven 4-week-old BALB/c mice (LASCO, Taipei, Taiwan) were divided into three groups as follows: group I (vaccination, n = 9), receiving 5 µg truncated E2 glycoprotein; group II (vaccination, n = 9), receiving 35 µg truncated E2 glycoprotein; and group III (control, n = 9), receiving phosphate-buffered saline (PBS). The mice immunized with 5 and 35 µg of truncated E2 glycoprotein served as the low-dose and high-dose groups, respectively. The mice were injected intraperitoneally twice at weeks 0 and 2 with 200 μL of the vaccine. Sera were obtained from each mouse for further determination at weeks 0, 2, 4, 6, and 8.

### Antibody analysis: total IgG, IgG_1_, and IgG_2a_

The specific serum titres of antibodies against BVDV were detected by indirect ELISA. Polystyrene flat-bottomed microtitre plates (ExtraGENE, Taichung, Taiwan) were coated with 1.25 μg/mL whole-virus antigen (BVDV/TW 2014) in coating buffer (15 mM Na_2_CO_3_, 35 mM NaHCO_3_, and 3 mM NaN_3_; pH 9.6) and left overnight at 4 °C. The optimal coating concentration and serum dilution were determined through chequerboard titration. The plates were washed three times with PBS containing Tween 20 (PBST), blocked with 1% BSA (KPL) in PBST at 37 °C for 1 h, and washed three times with PBST. Subsequently, 50 μL of adequately diluted mouse antiserum was added to the wells as the primary antibody, and the plates were incubated at 37 °C for 1.5 h. Thereafter, the plates were washed four times with PBST, and 100 μL of adequately diluted HRP-labelled goat anti-mouse IgG (KPL), anti-mouse IgG_1_ (polyclone, Bethyl Laboratories, Montgomery, TX, USA), or anti-mouse IgG_2a_ (polyclone, Bethyl Laboratories) was added as the secondary antibody. The plates were then incubated at 37 °C for 1 h and washed four times with PBST. Thereafter, 100 μL of TMB 2-Component Microwell Peroxidase Substrate (KPL) was added, and the colour reaction was performed for 10 min before TMB Stop Solution (KPL) was added to halt the reaction. Absorbance was measured at 450 nm by using a microplate reader (Anthos 2020; Anthos, Cambridge, UK).

### Lymphocyte proliferation assay

Four weeks post-immunization, the lymphocyte proliferation assay was performed using the Cell Titer 96 AQ_ueous_ One Solution Cell Proliferation Assay (MTS; Promega). Splenocytes from four randomly selected mice per group were seeded into a 96-well flat-bottomed plate (1 × 10^6^ cells per well) and co-cultured with truncated E2 glycoprotein (5 μg/mL) in RPMI-1640 supplemented with 10% FBS, and maintained at 37 °C in a humidified 5% CO_2_ atmosphere for 72 h. In addition, cell-free media containing complete RPMI-1640 were run in parallel to the test. The cells alone served as the negative control, and the potent mitogen concanavalin A (ConA; Sigma-Aldrich, MO, USA) was used as a positive control for a final concentration of 2 μg/mL. Each splenocyte sample was evaluated in triplicate. MTS (3-(4,5-Dimethylthiazol-2yl)-5-(3-carboxymethoxyphenyl)-2-(4-sulfophenyl)-2 H-tetrazolium) was added to each well and then incubated for 4 h at 37 °C under 5% CO_2_. The absorbance at 490 nm was measured. The stimulation index was determined as follows: (optical diffraction [OD] of treatment − OD of background)/(OD of the negative control − OD of background).

### T lymphocytes sub-populations assay

Four mice were used to determine the frequency of CD4^+^ and CD8^+^ T cells for each test group at 4 weeks post-immunization. Briefly, splenocytes from each group’s mice were isolated by disrupting the spleens using a 3-mL syringe barrel in RPMI-1640 (Gibco, Grand Island, NY, USA). After the spleen homogenates were passed through a 100-μm cell strainer and treated in ACK lysing buffer (Thermo Fisher Scientific, Waltham, MA, USA) to lyse erythrocytes, the cells were washed twice and resuspended in PBS containing 1% albumin from bovine serum (1 × 10^7^ cells/mL). Isolated splenocytes (10^6^ cells) were incubated with FITC-conjugated rat anti-mouse CD4^**+**^ and PE-conjugated rat anti-mouse CD8^**+**^ (BD Pharmingen, San Jose, CA) at 4 °C for 30 min in the dark. Subsequently, the cells were washed three times with PBS, and 50,000 cells, according to the proportion of CD4^+^ and CD8^+^ T cells, were characterized by flow cytometry using a FACScalibur instrument (BD Biosciences).

### Cytokine assay

Cytokine levels in serum collected from immunized mice at weeks 0, 2, and 4, respectively, were detected using commercial interleukin (IL)-12 p70 (ab119531), interferon-gamma (IFN-γ; ab100689), and IL-4 (ab100710) ELISA kits (Abcam, Cambridge, MA, USA), in accordance with the manufacturer’s protocol. The concentration of each cytokine was calculated using the plotted standard curve.

### Statistical analysis

All data were analyzed using SAS statistical software (v. 9.0, Cary, NC, USA). For the mouse experiments, differences among the treatments at each time point were analyzed using analysis of variance and Duncan’s multiple comparisons. Different letters (a, b, c) on the bar indicate significant (*P* < 0.05) between-group differences at the same time point.

## Data availability statement

The datasets generated and/or analyzed during the current study are available in the Uniprot repository, accession number C4NF75.

## Supplementary Information

Supplementary Figure 1.

## References

[1] Fulton RW (2013). Host response to bovine viral diarrhea virus and interactions with infectious agents in the feedlot and breeding herd. Biologicals.

[2] Rebhun WC (1989). Thrombocytopenia associated with acute bovine virus diarrhea infection in cattle. J. Vet. Intern. Med..

[3] Brock KV (2003). The persistence of bovine viral diarrhea virus. Biologicals.

[4] Houe H (1999). Epidemiological features and economical importance of bovine virus diarrhoea virus (BVDV) infections. Vet. Microbiol..

[5] Richter V (2017). A systematic worldwide review of the direct monetary losses in cattle due to bovine viral diarrhoea virus infection. Vet. J..

[6] Schweizer M, Peterhans E (2014). Pestiviruses. Annu. Rev. Anim. Biosci..

[7] Decaro N (2011). Atypical pestivirus and severe respiratory disease in calves. Europe. Emerg. Infect. Dis..

[8] McClurkin AW, Bolin SR, Coria MF (1985). Isolation of cytopathic and noncytopathic bovine viral diarrhea virus from the spleen of cattle acutely and chronically affected with bovine viral diarrhea. J. Am. Vet. Med. Assoc..

[9] Moennig V, Liess B (1995). Pathogenesis of intrauterine infections with bovine viral diarrhea virus. Vet. Clin. N. Am. Food Anim. Pract..

[10] Yeşilbağ K, Alpay G, Becher P (2017). Variability and global distribution of subgenotypes of bovine viral diarrhea virus. Viruses.

[11] Moennig V, Becher P (2018). Control of bovine viral diarrhea. Pathogens.

[12] Rodning SP (2010). Comparison of three commercial vaccines for preventing persistent infection with bovine viral diarrhea virus. Theriogenology.

[13] Koethe S (2020). A synthetic modified live chimeric marker vaccine against BVDV-1 and BVDV-2. Vaccines.

[14] Klimowicz-Bodys MD (2021). Antibody response to a live-modified virus vaccine against bovine viral diarrhoea in dairy cattle in a field trial. Vaccines.

[15] Al-Kubati AAG, Hussen J, Kandeel M, Al-Mubarak AIA, Hemida MG (2021). Recent advances on the bovine viral diarrhea virus molecular pathogenesis, immune response, and vaccines development. Front. Vet. Sci..

[16] Donofrio G, Bottarelli E, Sandro C, Flammini CF (2006). Expression of bovine viral diarrhea virus glycoprotein E2 as a soluble secreted form in a mammalian cell line. Clin. Vaccine Immunol..

[17] Nogarol C (2017). Pestivirus infection in cattle dairy farms: E2 glycoprotein ELISA reveals the presence of bovine viral diarrhea virus type 2 in northwestern Italy. BMC Vet. Res..

[18] de Felipe P, Ryan MD (2004). Targeting of proteins derived from self-processing polyproteins containing multiple signal sequences. Traffic.

[19] de Felipe P (2006). E unum pluribus: multiple proteins from a self-processing polyprotein. Trends Biotechnol..

[20] Gupta R, Brunak S (2002). Prediction of glycosylation across the human proteome and the correlation to protein function. Pac. Symp. Biocomput..

[21] Fulton RW, Cook BJ, Payton ME, Burge LJ, Step DL (2020). Immune response to bovine viral diarrhea virus (BVDV) vaccines detecting antibodies to BVDV subtypes 1a, 1b, 2a, and 2c. Vaccine.

[22] Platt R, Kesl L, Guidarini C, Wang C, Roth JA (2017). Comparison of humoral and T-cell-mediated immune responses to a single dose of Bovela® live double deleted BVDV vaccine or to a field BVDV strain. Vet. Immunol. Immunopathol..

[23] Meyers G (2007). Bovine viral diarrhea virus: prevention of persistent fetal infection by a combination of two mutations affecting E^rns^ RNase and N^pro^ protease. J. Virol..

[24] Wernike K (2018). The occurrence of a commercial Npro and Erns double mutant BVDV-1 live-vaccine strain in newborn calves. Viruses.

[25] Bolin SR, Matthews PJ, Ridpath JF (1991). Methods for detection and frequency of contamination of fetal calf serum with bovine viral diarrhea virus and antibodies against bovine viral diarrhea virus. J. Vet. Diagn. Invest..

[26] Makoschey B, van Gelder PT, Keijsers V, Goovaerts D (2003). Bovine viral diarrhoea virus antigen in foetal calf serum batches and consequences of such contamination for vaccine production. Biologicals.

[27] Newcomer BW, Walz PH, Givens MD, Wilson AE (2015). Efficacy of bovine viral diarrhea virus vaccination to prevent reproductive disease: a meta-analysis. Theriogenology.

[28] Platt R, Coutu C, Meinert T, Roth JA (2008). Humoral and T cell-mediated immune responses to bivalent killed bovine viral diarrhea virus vaccine in beef cattle. Vet. Immunol. Immunopathol..

[29] Stevens ET (2009). The induction of a cell-mediated immune response to bovine viral diarrhea virus with an adjuvanted inactivated vaccine. Vet. Ther..

[30] Sozzi E (2020). Cross-reactivity antibody response after vaccination with modified live and killed bovine viral diarrhoea virus (BVD) vaccines. Vaccines.

[31] Walz PH (2018). Comparison of reproductive protection against bovine viral diarrhea virus provided by multivalent viral vaccines containing inactivated fractions of bovine viral diarrhea virus 1 and 2. Vaccine.

[32] Lo YT (2021). Expression and immunogenicity of secreted forms of bovine ephemeral fever virus glycoproteins applied to subunit vaccine development. J. Appl. Microbiol..

[33] O'Flaherty R (2020). Mammalian cell culture for production of recombinant protein: a review of the critical steps in their biomanufacturing. Biotechnol. Adv..

[34] Chen Q, He F, Kwang J, Chan JK, Chen J (2012). GM-CSF and IL-4 stimulate antibody responses in humanized mice by promoting T, B, and dendritic cell maturation. J. Immunol..

[35] Balenović M, Savić V, Ekert Kabalin A, Jurinović L, Ragland WL (2011). Abundance of IFN-α and IFN-γ gene transcripts and absence of IL-2 transcripts in the blood of chickens vaccinated with live or inactivated NDV. Acta Vet. Hung..

[36] Doherty PC (1997). Effector CD4^+^ and CD8^+^ T-cell mechanisms in the control of respiratory virus infections. Immunol. Rev..

[37] Luke GA, Ryan MD (2013). The protein coexpression problem in biotechnology and biomedicine: virus 2A and 2A-like sequences provide a solution. Future Virol..

[38] Minskaia E, Nicholson J, Ryan MD (2013). Optimisation of the foot-and-mouth disease virus 2A coexpression system for biomedical applications. BMC Biotechnol..

[39] Chung YC (2018). Recombinant E2 protein enhances protective efficacy of inactivated bovine viral diarrhea virus 2 vaccine in a goat model. BMC Vet. Res..

[40] Reed LJ, Muench H (1938). A simple method of estimating fifty percent endpoints. Am. J. Epidemiol..

[41] Kosinova E, Psikal I, Robesova B, Kovarcik K (2007). Real-time PCR for quantitation of bovine viral diarrhea virus RNA using SYBR green I fluorimetry. Vet. Med..

