# Supplementary material for: Immunogenicity of a secreted, C-terminally truncated, form of bovine viral diarrhea virus E2 glycoprotein as a potential candidate in subunit vaccine development
Source: Sci Rep. 2023 Jan 6;13:296. doi: 10.1038/s41598-022-26766-y (PMC9818055; doi:10.1038/s41598-022-26766-y)

**Supplementary Information**

**Supplementary Figure 1 Legend:**

Western blot analyses of transfected cell extracts. Samples of cellular extracts were prepared and analyzed (12% SDS–PAGE) prior to the transfer, as described in methods. Membranes were probed with the mouse anti-V5 monoclonal antibody (1:8000) (Supplementary Figure 1A) or mouse anti-BVDV antiserum (1:4000) (Supplementary Figure 1B) as primary antibody. Supplementary Figure 1A and 1B are original and unprocessed images of Figure 2A.

The additional band at 25 kDa could be a cellular degradation product of ‘uncleaved’ [GFP-2A-BVDV-E2-V5] in the cytoplasm, or, degradation of [BVDV-E2-V5] in the endoplasmic reticulum or the Golgi apparatus.

Supplementary Figure 1A. The left side of image are two different size of protein marker.


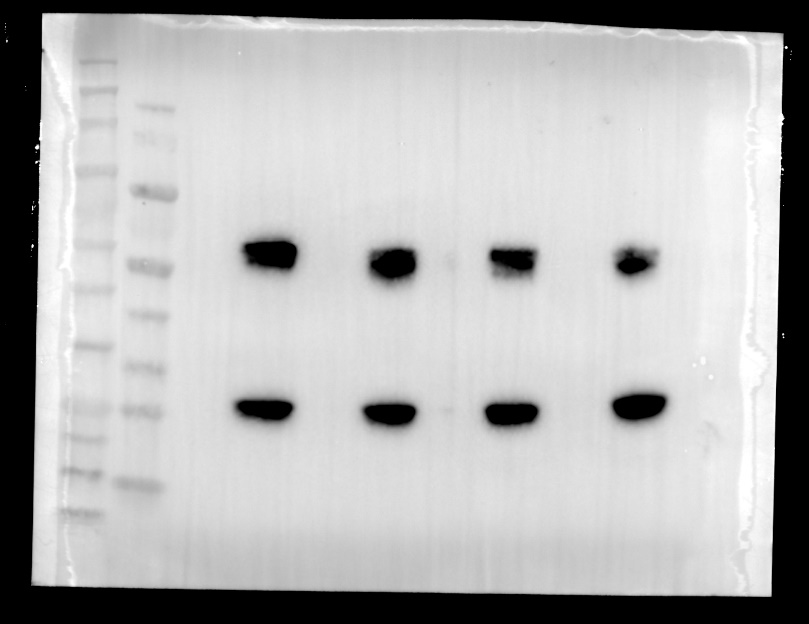


Supplementary Figure 1B. The left side of image are three different size of protein marker.


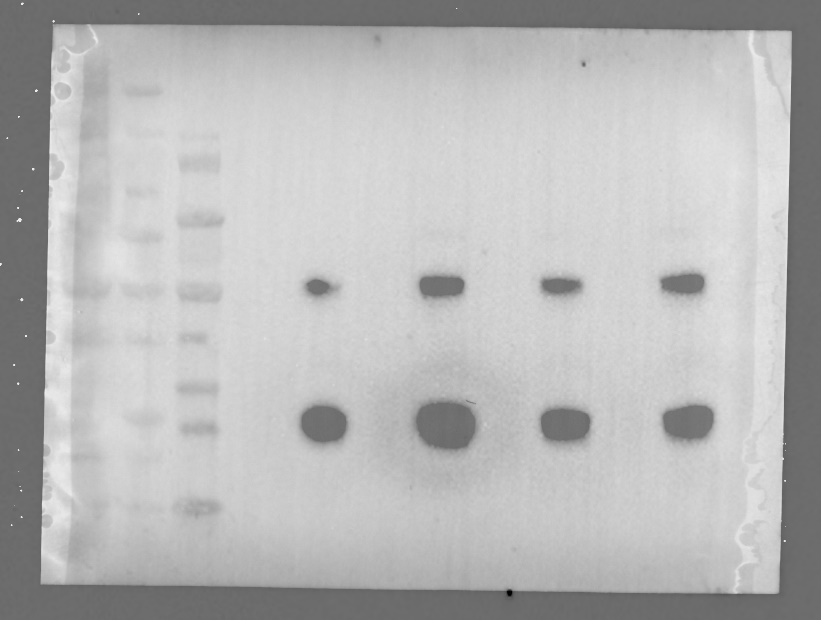

Supplement: Supplementary file 1 — Supplementary Figure 1. [file 41598_2022_26766_MOESM1_ESM.docx]
